# Carbon-Based Band Gap Engineering in the h-BN Analytical Modeling

**DOI:** 10.3390/ma13051026

**Published:** 2020-02-25

**Authors:** Mohammad Taghi Ahmadi, Ahmad Razmdideh, Seyed Saeid Rahimian Koloor, Michal Petrů

**Affiliations:** 1 Division of Computational Physics, Institute for Computational Science, Ton Duc Thang University, Ho Chi Minh City 758307, Vietnam; 2 Faculty of Electrical and Electronics Engineering, Ton Duc Thang University, Ho Chi Minh City 758307, Vietnam; 3 Nano-electronic Research Group, Physics Department, Faculty of Science, Urmia University, Urmia 57147, Iran; ahmadi.ph@gmail.com; 4 Institute for Nanomaterials, Advanced Technologies and Innovation, Technical University of Liberec, Studentska 2, 461 17 Liberec, Czech Republic; s.s.r.koloor@gmail.com

**Keywords:** hoping energy, BC_2_N band gap engineering, BC_2_N band energy, tight binding

## Abstract

The absence of a band gap in graphene is a hindrance to its application in electronic devices. Alternately, the complete replacement of carbon atoms with B and N atoms in graphene structures led to the formation of hexagonal boron nitride (h-BN) and caused the opening of its gap. Now, an exciting possibility is a partial substitution of C atoms with B and N atoms in the graphene structure, which caused the formation of a boron nitride composite with specified stoichiometry. BC_2_N nanotubes are more stable than other triple compounds due to the existence of a maximum number of B–N and C–C bonds. This paper focused on the nearest neighbor’s tight-binding method to explore the dispersion relation of BC_2_N, which has no chemical bond between its carbon atoms. More specifically, the band dispersion of this specific structure and the effects of energy hopping in boron–carbon and nitrogen–carbon atoms on the band gap are studied. Besides, the band structure is achieved from density functional theory (DFT) using the generalized gradient approximations (GGA) approximation method. This calculation shows that this specific structure is semimetal, and the band gap energy is 0.167 ev.

## 1. Introduction

Nowadays, frontier technologies, especially in the semiconductor and battery industries, are improving material scaling to enhance the efficiency of their products. In particular, foiling unwanted transport between anode and cathode in the batteries is engaged by material performance. One the other hand, from the semiconductor-based point of view, one of the available techniques to progress the artifact is hidden in the BN material performance, which can be controlled by supplementary carbon atoms. For example, in the form of BC_2_N, the band gap can be controlled effortlessly. On the other hand, the BC_2_N allotropes as negative electrodes and anchoring materials for Li-S has been reported [1,2]. Since an increase in the numbers and efficiency of transistors diminishes the cost and leads to computers with faster processing ability, the number of such transistors on a microchip is commonly on the rise. Given this, it seems very cost effective to reduce the size of transistors [3,4]. However, this minimizing process will be finally stopped due to the need for developments in electronic commerce; thus, alternative technologies are needed [3]. Hence, the search for a matter with good nanoelectronics and optoelectronics functions is an important issue [5]. Alternative technology should solve past problems and have economic justification. Hereupon, to deal with this problem, the nanotechnology was integrated with electronic industry, and therefore, nanoelectronics was born [6].

Over the past 50 years, the use of Moore’s law has been effective in relieving silicon-based electronic components for nanoelectronics [7] and optoelectronics [8]. The two-dimensional graphene, due to its extraordinary electronic properties including high carrier velocity and mechanical strengths, is a good candidate for electronic devices [9,10]. However, the lack of a gaping band prevents it from being used in field effect transistors (MOSFET) [11], and this has led to the use of two-dimensional minerals such as graphite or single-layer boron nitride. Moreover, boron nitride has a structural similarity to carbon (C) and appears in different crystalline forms [12]. Besides, unlike graphene, due to the specific accumulation of monolayers by boron nitride, it is rarely available. In addition, because of the polarity of the bonds between B–N atoms in forming a multilayer of boron nitride, the whole system will be stable [13]. Boron nitride is known as a semiconductor with a large gap of 5 to 6 electron volts due to its wideband semiconducting behavior [14,15]. Furthermore, an interesting possibility is the substitution of the C atoms with B and N atoms in the sites of the honeycomb structure of graphene [16], which leads to the formation of a triplet-stoichiometric compound.

In 2006, the structure and electronic properties of B_X_C_Y_N_Z_ with stoichiometry ratios and various formations of the Ab Initio calculations were studied [17]. The results show that the amount of stability and energy gap depends on the array of atoms and their stoichiometry properties and is not related to the dimensions of the cell [17]. The structural similarity between graphite and hexagonal boron nitride became a motive for synthesizing the alloys of this compound [18]. On the one hand, the electronic properties of B_X_C_Y_N_Z_ are similar to graphite in a semimetal manner [19], and the insulator attitude is similar to that of boron nitride based on the doping with boron and nitrogen atoms [20]; therefore, the location and the amount of impurities affect the structure’s stability. Meanwhile, the structure of BC_2_N is zigzag-bonded, and it makes separate units of graphite and island-like boron nitride [21]. Therefore, determining the preferable structure of the BC_2_N monolayer, configurational strain, and the atomic bond has a considerable role [22]. 

It was theoretically predicted that through the use of first-principles calculation and CVD (chemical vapor deposition) synthesis in the presence of BC_13_, CC1_4_, N_2_, and H_2_ based on XRD analysis, the existence of hexagonal layers by a 2.44 angstrom lattice constant with a 3.40 angstrom interlayer space has been confirmed [23]. Although the X-ray data fail to provide the atomic arrangement in planar form, they do designate that the boron, carbon, and nitrogen atom arrangement in the BC_2_N is in the form of sp^2^-type bonding [24]. As a result, it has been anticipated that the atoms will be arranged in planes of hexagonal rings similar to graphite, with weak interplanar interactions [25]. At this point, it remains vague whether BC_2_N functions as a semiconductor or a semimetal [26]. Furthermore, presumably the struggle between strain and bonding energy leads to the single-layered BC_2_N structure. Hence, whether BC_2_N is a semiconductor or a semimetal is an issue of controversy [26]. Therefore, band-gap engineering is a traditional and an influential technique in adjusting a new energy gap to a desirable value and designing new semiconductor materials [27]. To this end, there are diverse methods, including atom arrangement change, doping, and applying electric fields, among others.

**Figure 1:**
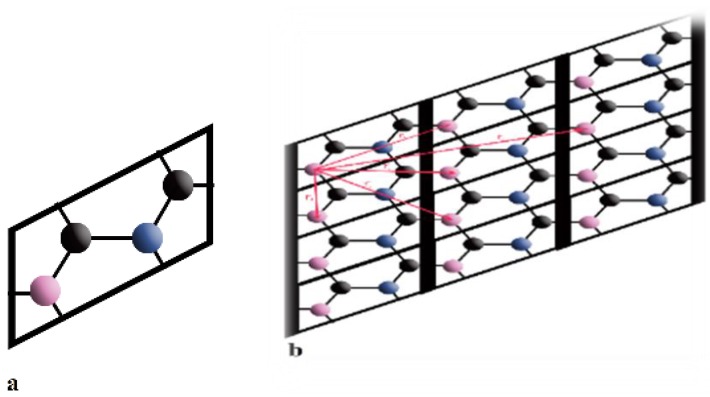
(**a**) Unit cell of BC_2_N; (**b**) ultimate Structure of BC_2_N.

The present study is concerned with the analytical investigation of energy dispersion through the use of the Nearest Neighbor Tight Binding (NNTB) approximation method [28] for BC_2_N material in which boron and nitrogen are both bonded with three carbons in a way that none of the carbons are bonded with each other, as shown in Figure 1. Another feature of this structure is that it has no symmetry in the space group, but the angle between the straight lines of combined boron and nitrogen atoms is 60 degrees.

## 2. Modeling

The NNTB approximation method is used to model the band structure of BC_2_N, based on Figure 1a [28]. In this regard, one-way is used to investigate how the electron–electron interaction in the structure of crystalline solves a separable Schrödinger equation, and this model is achieved by means of a time-independent Schrödinger equation. The outcome of the time-independent equation (Schrödinger) is written in the form of

(1) $$E\emptyset_{0}=\left[h\left(k\right)\right]\emptyset_{0}$$

where $\emptyset_{0}$ is the wave function, and h(k) is the matrix equation as

(2) $$h\left(k\right)=\overset{4}{\underset{m=1}{\sum }}H_{mn}e^{ik\left(d_{m}-d_{n}\right)}$$

In Equation (2), $\;H_{mn}$ stands for the Hamiltonian matrix equation, k is the wave vector, and $\;d_{m}$ and $d_{n}$ are the displacements of the m^th^ and n^th^ unit cells from the origin, respectively. On the other hand, for this special model, no chemical bonds between carbon–carbon bonds are considered. Now, in this case, B assigns $E_{oC}$, $E_{oB}$, and $E_{oN}$ to the onsite energies of carbon, boron, and nitrogen atoms, respectively, and the overlapping energy between the two atoms of B–C and N–C are designated by $t_{bc}=t\;$ and $t_{cn}=t_{p}$, respectively. Thus, Equation (2) can be presented via:

(3) $$h(k)\;=\;\left[\begin{array}{cc} \begin{array}{cc} h_{1} & 0\\ 0 & h_{1} \end{array} & \begin{array}{cc} t_{p} & h_{2}\\ h_{2} & h_{4} \end{array}\\ \begin{array}{cc} t_{p} & h_{3}\\ h_{2} & h_{4} \end{array} & \begin{array}{cc} h_{1} & 0\\ 0 & h_{1} \end{array} \end{array}\right]$$

where h_1_ = (E_0_ + 2 × E_0_ × (cos(k.r_1_) + cos(k.r_2_)))E, h_2_ = t + t × (cos(k.r_1_)), h_3_ = t_p_ + t_p_ × (cos(k.r_1_)), h_4_ = t × (cos(k.r_2_)), k = k_x_ i + k_y_ j, r_1_ = 3 × a_0_ i, and r_2_ = 3a_0_i +$\;\sqrt{3}$ a_0_j. For interpretation purposes, it is assumed that Figure 1b is an ideal hexagonal shape and has an equal bond length of a_0_ = 1.446 Å between boron and nitrogen atoms. Therefore, to define the energy *eigenvalues* in the matrix form, the energy matrix is diagonalized by

(4) $$\det\left[\begin{array}{cc} \begin{array}{cc} h_{1} & 0\\ 0 & h_{1} \end{array} & \begin{array}{cc} t_{p} & h_{2}\\ h_{2} & h_{4} \end{array}\\ \begin{array}{cc} t_{p} & h_{3}\\ h_{2} & h_{4} \end{array} & \begin{array}{cc} h_{1} & 0\\ 0 & h_{1} \end{array} \end{array}\right]\;=\;0.$$

The energy eigenvalues based on the Schrödinger equation solution lead to the following four energy dispersion relations for this particular BC2N structure due to the introduced unit cell, as shown in Figure 1a.

(5) $$\begin{array}{l} E_{1,2}=\left(2\cos\left(a\right)+2\cos\left(b\right)+1\right)E_{0}\pm \frac{\sqrt{2}}{2}((2t^{3}t^{\prime }\cos{\left(a\right)}^{2}\cos{\left(b\right)}^{2}\\ +4t^{3}t^{\prime }\cos{\left(a\right)}^{2}\cos\left(b\right)+4t^{3}t^{\prime }\cos\left(a\right)\cos{\left(b\right)}^{2}\\ +8t^{3}t^{\prime }\cos\left(a\right)\cos\left(b\right)+4t^{2}{t^{\prime }}^{2}\cos{\left(a\right)}^{2}\cos\left(b\right)+8t^{2}{t^{\prime }}^{2}\cos\left(a\right)\cos\left(b\right)\\ +2t{t^{\prime }}^{3}+{t^{\prime }}^{4}+3t^{2}{t^{\prime }}^{2}+t^{4}+t^{4}(\cos{\left(a\right)}^{4}\times 4t^{4}\cos{\left(a\right)}^{3}+6t^{4}\cos{\left(a\right)}^{2}\\ +4t^{4}\cos\left(a\right)+t^{4}\cos{\left(b\right)}^{4}+2t^{4}\cos{\left(b\right)}^{2}-2t^{3}t^{\prime }+4t{t^{\prime }}^{3}\cos\left(a\right)\\ +2t{t^{\prime }}^{3}\cos{\left(a\right)}^{2}t^{3}+4t^{2}{t^{\prime }}^{2}\cos\left(b\right)+8t^{2}{t^{\prime }}^{2}\cos\left(a\right)-2t^{2}{t^{\prime }}^{2}\cos{\left(b\right)}^{2}\\ +4t^{2}{t^{\prime }}^{2}{\left(\cos\left(a\right)\right)}^{2}+8t^{2}{t^{\prime }}^{2}{\left(\cos\left(a\right)\right)}^{2}+2t{t^{\prime }}^{2}{\left(\cos\left(b\right)\right)}^{2}-8t^{3}t^{\prime }\cos\left(a\right)\\ +4t^{3}t^{\prime }\cos\left(b\right)+t^{2}{t^{\prime }}^{2}{\left(\cos\left(a\right)\right)}^{4}-2t^{3}t^{\prime }{\left(\cos\left(a\right)\right)}^{4}-8t^{3}t^{\prime }{\left(\cos\left(a\right)\right)}^{3}\\ -12t^{3}t^{\prime }{\left(\cos\left(a\right)\right)}^{2}+2t^{4}{\left(\cos\left(a\right)\right)}^{2}{\left(\cos\left(b\right)\right)}^{2}\\ +2t^{4}{\left(\cos\left(a\right)\right)}^{2}{\left(\cos\left(b\right)\right)}^{2}{)}^{\frac{1}{2}}+tt^{\prime }+t^{2}{\left(\cos\left(a\right)\right)}^{2})+t^{2}{\left(\cos\left(b\right)\right)}^{2}\\ +t^{2}+{t^{\prime }}^{2}+2t^{2}\cos\left(a\right)+2tt^{\prime }\cos\left(a\right)+tt^{\prime }{\left(\cos\left(a\right)\right)}^{2}{)}^{\frac{1}{2}} \end{array}$$

(6) $$\begin{array}{l} E_{3,4}=\left(2\times \cos\left(a\right)+2\cos\left(b\right)+1\right)\times E_{0}\pm \frac{\sqrt{2}}{2}\times (tt^{\prime }\pm 2t^{3}t^{\prime }\cos{\left(a\right)}^{2}\cos{\left(b\right)}^{2}\\ +4t^{3}t^{\prime }\cos{\left(a\right)}^{2}\cos\left(b\right)+4t^{3}t^{\prime }\cos\left(a\right)\cos{\left(b\right)}^{2}\\ +8t^{3}t^{\prime }\cos\left(a\right)\cos\left(b\right)+4t^{2}{t^{\prime }}^{2}\cos{\left(a\right)}^{2}\cos\left(b\right)+8t^{2}{t^{\prime }}^{2}\cos\left(a\right)\cos\left(b\right)\\ +2t{t^{\prime }}^{3}+{t^{\prime }}^{4}+3t^{2}{t^{\prime }}^{2}+t^{4}+t^{4}(\cos{\left(a)\right)}^{4}\times 4t^{4}(\cos{\left(a)\right)}^{3}\\ +6t^{4}\cos{\left(a\right)}^{2}+4t^{4}\cos\left(a\right)+t^{4}\cos{\left(b\right)}^{4}+2t^{4}\cos{\left(b\right)}^{2}-2t^{3}t^{\prime }\\ +4t{t^{\prime }}^{3}\cos\left(a\right)+2t{t^{\prime }}^{3}\cos{\left(a\right)}^{2}+4t^{2}{t^{\prime }}^{2}\cos\left(b\right)\\ +8t^{2}{t^{\prime }}^{2}\cos\left(a\right)-2t^{2}{t^{\prime }}^{2}\cos{\left(b\right)}^{2}+4t^{2}{t^{\prime }}^{2}{\left(\cos\left(a\right)\right)}^{2}\\ +8t^{2}{t^{\prime }}^{2}{\left(\cos\left(a\right)\right)}^{2}+2t{t^{\prime }}^{2}{\left(\cos\left(b\right)\right)}^{2}-8t^{3}t^{\prime }\cos\left(a\right)+4t^{3}t^{\prime }\cos\left(b\right)\\ +t^{2}{t^{\prime }}^{2}{\left(\cos\left(a\right)\right)}^{4}-2t^{3}t^{\prime }{\left(\cos\left(a\right)\right)}^{4}-8t^{3}t^{\prime }{\left(\cos\left(a\right)\right)}^{3}\\ -12t^{3}t^{\prime }{\left(\cos\left(a\right)\right)}^{2}+2t^{4}{\left(\cos\left(a\right)\right)}^{2}{\left(\cos\left(b\right)\right)}^{2}+4t^{4}\cos\left(a\right){\left(\cos\left(b\right)\right)}^{2}{)}^{\frac{1}{2}}\\ +tt^{\prime }+t^{2}{\left(\cos\left(a\right)\right)}^{2}+t^{2}{\left(\cos\left(b\right)\right)}^{2}+t^{2}+{t^{\prime }}^{2}\\ +2t^{2}\cos\left(a\right)+2tt^{\prime }\cos\left(a\right)+tt^{\prime }{\left(\cos\left(a\right)\right)}^{2}{)}^{\frac{1}{2}} \end{array}$$

**Figure 2:**
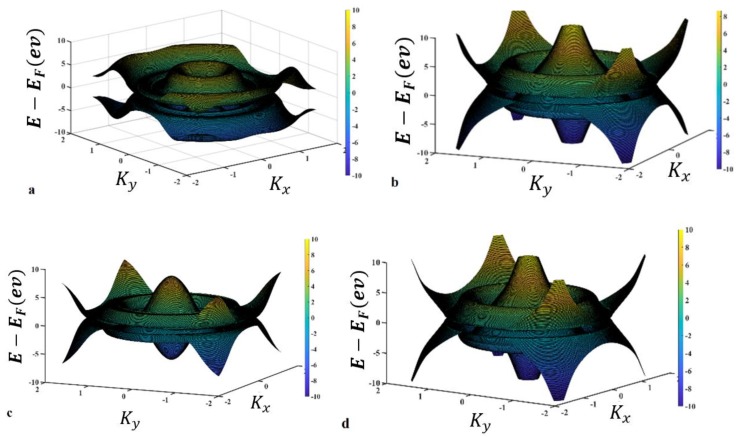
(**a**) One of the acceptable respondents from the energy determinant with a three-dimensional band structure scheme mainly consisting of BC_2_N; (**b**) The band gap resulting from the Equation (3) is due to E_1_ is 2.416 ev; (**c**) The band gap corresponding to Equation (4), namely E_2_, is about 1.212 ev; (**d**) On the other hand, the band gap resulting from Equation (5) E_3_ is 0.7182 ev. Finally, as shown, through solving Equation (6), it is found that the fourth possible band gap E_4_ is 1.3254 ev.

To elucidate the variation of the electronic band structure in BC_2_N, we generated an E–K relation according to Figure 1. The variation in the band gap together with solving Equations (5) and (6), which are named dispersion relation equations, is shown in Figure 2.

## 3. Result and Discussion

**Figure 3:**
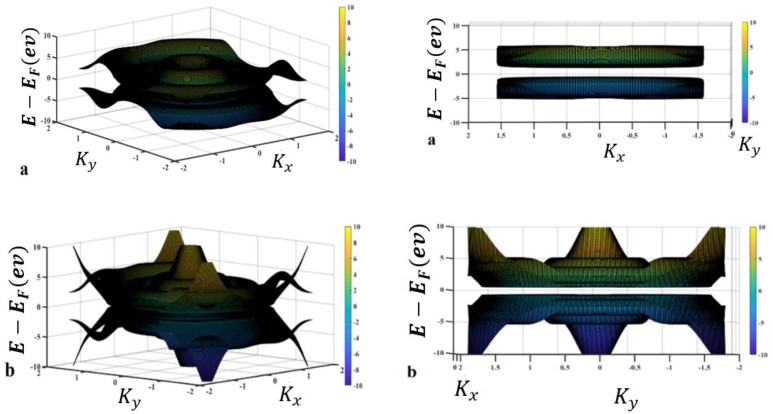

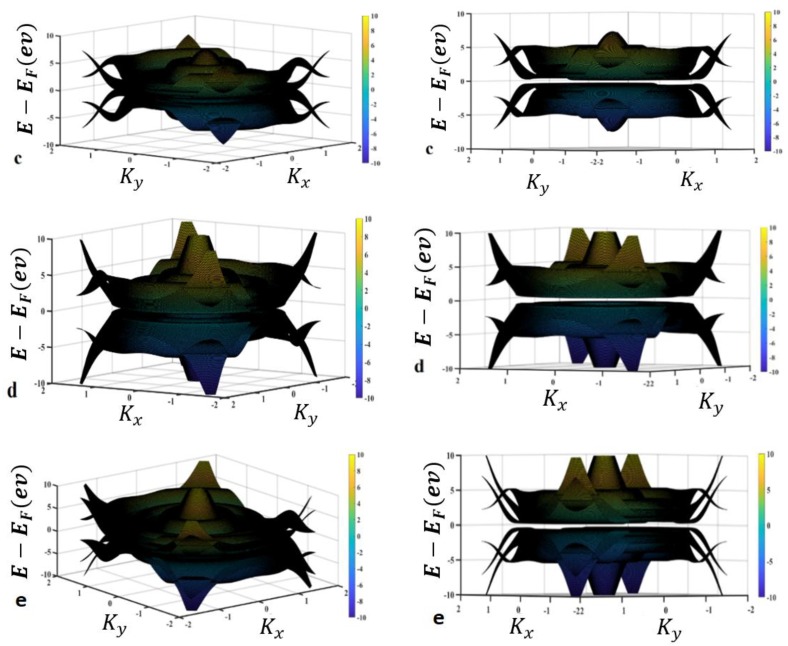
(**a**) Dispersion relation plotted for E_1_, which indicates a band gap = 2.312 ev; (**b**) dispersion relation plotted for both E_1_, and E_2_, in which the minimum band gap is found to be 1.1988 ev; (**c**) dispersion relations for both E_1_ and E_3_ are compared, and a minimum of about 0.7182 ev is reported; (**d**) dispersion relations for both E_1_ and E_4_ are compared, and a minimum band gap of about 1.304 ev is shown; (**e**) dispersion relation results for E_1_, E_2_, E_3_, and E_4_ are compared, and a minimum band gap of about 0.7182 ev is addressed. Note: In the right-hand side of each figure, a two-dimensional view is presented with the same label.

It is found that there are four answers due to the variables of wave vector and energy. The first answer is E_1_ (Figure 2a), which indicates a gap of 2.416 ev; the second answer is E_2_ with a 1.212 ev band gap (Figure 2b), the band gap due to the third answer, E_3_, is equal to 0.7182 ev (Figure 2c), and finally, the fourth answer E_4_ with a 1.3254 ev band gap (Figure 2d). The band structure that is plotted using E_1_, E_2_, E_3_, and E_4_, resulted from Equations (5) and (6), which demonstrates the suggested model, as shown in Figure 3. In the other words, the dispersion relation (Equation (6)) leads to the formation of the band structure, as shown in Figure 2 and Figure 3.

As shown in Figure 3, the possible band gaps based on the presented mode are analyzed and plotted in three-dimensional (right-hand side figures) and two-dimensional (left-hand side figures) forms in comparison with each other starting with E_1_ as shown in Figure 2a, followed by the accumulation of band structures E_2_ to E_4._

**Figure 4:**
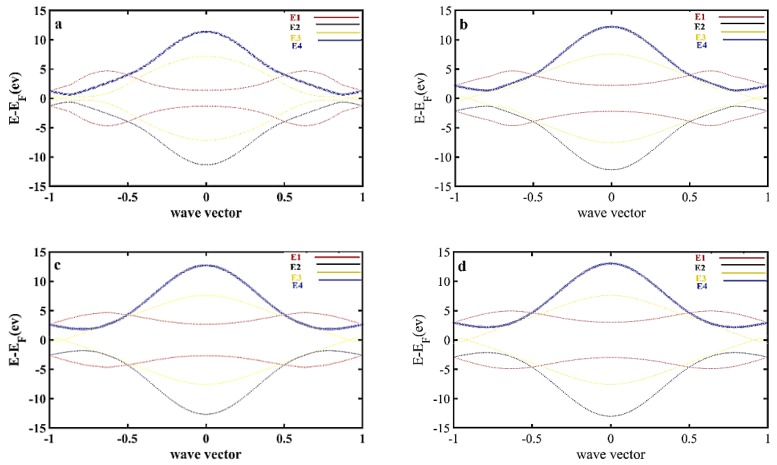
(**a**) t = 2.59 ev, tp = 1 ev; (**b**) for t = 2.59 ev, tp = 2 ev; (**c**) t = 2.59 ev, tp = 2.5 ev; (**d**) t = 2.5 ev, tp = 2.83 ev.

In the technology application, the ultimate lattice structure is under unwanted strain and stresses that cause band energy variation. To analyze this annoying effect, one of the imaginable methods is the overlap energy variation investigation, which can be realized in the form of lattice parameter variation. Therefore, the effect of overlap energy between carbon–nitrogen and carbon–boron atoms as a disparity between the highest and lowest energy levels is considered as shown in Figure 4. In the other words, the reduction in the lattice parameter could cause an increase in the spatial overlap of the orbitals. On the other hand, the increased anti-bonding appears to be larger than the increased bonding. For simplification purposes, the hopping energy of boron–carbon and nitrogen–carbon is shown in terms of t and t_p_, respectively. As indicated in Figure 3, the energy overlaps between B–C, namely, t_p_, is fixed at 2.59 ev, and the energy overlaps between C–N, namely t, is changed. Therefore, band gap energies of about a) E_1_ = 2.594 ev, b) E_1_ = 4.266 ev, c) E_1_ = 5.11 ev, and d) E_1_ = 5.6 ev are reported. These results show that in accordance with the increase in t_p_, the band gap increases.

**Figure 5:**
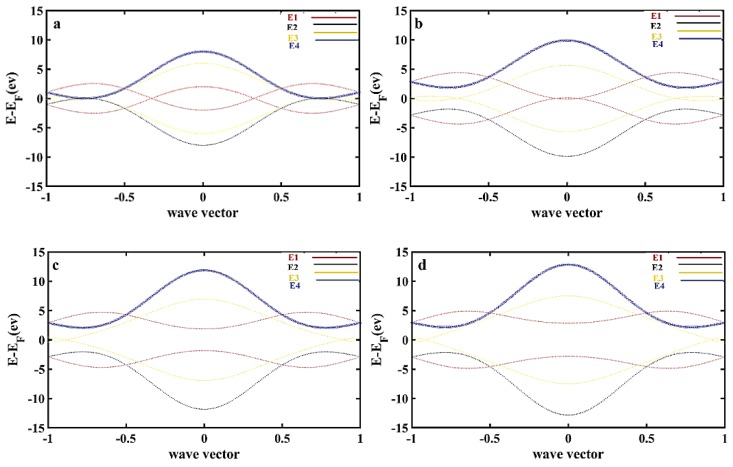
(**a**) t = 1 ev, tp = 1 ev; (**b**) t = 1 ev, tp = 2.83 ev; (**c**) t = 2 ev, tp = 2.83 ev; (**d**) t = 2.5 ev, tp = 2.83 ev.

On the other hand, via changing the overlapping energy between nitrogen and carbon, namely t, an overlap between energy bands is reported. Besides, the effect of overlap energy between carbon and boron is plotted, as shown in Figure 5.

It is concluded that any variation in the overlap energy indicates a direct effect on the BC_2_N band structure, which can be explained by an applied quantum confinement effect in the energy matrix. In the presented model, it can be assumed that the quantum confinement effect is buried in the overlap energy variation. On the other hand, density functional theory (DFT) and tight binding (TB) methods have been employed intensively in the material property investigation [29,30,31,32]. The DFT illustrates good agreement with experimental results; however, it is computationally very expensive. Therefore, its application has been limited to a small amount of atom calculations [32]. In contrary, the TB method in the band structure calculation without self-consistent progressions needs smaller amounts of computational possessions. Therefore, TB models have been applied in the large structure (up to millions of atoms) investigations [29]. In addition, the TB model often leads to analytical terminologies that improve the logical investigation of material properties; consequently, in this research, a TB model is being implemented. On the other hand, to simulate the DFT, we use an OPENMX3.8.5-open source computer code with a lattice constant of about a = b = 5.0503 Å and c = 15.2997 Å. In addition, for the linear combination of the pseudo-atomic localized basis set, 150 Ry cutoff energy and 10 × 10 × 1 k-point are used. The basis set function is B7.0-s2p2d1, N6.0-s2p2d1, and C6.0-s2p2d1 for boron, nitrogen, and carbon, respectively. The first symbol shows the chemical name together with the cutoff radius and the initial orbitals, which is shown via the last set. All data are achieved using generalized gradient approximations (GGA) and the PBE exchange-correlation functional.

**Figure 6:**
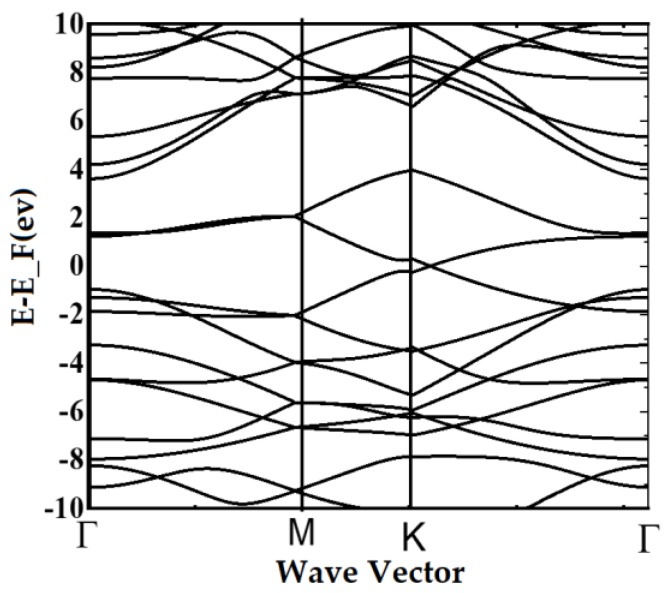
The band structure of BC_2_N.

**Figure 7:**
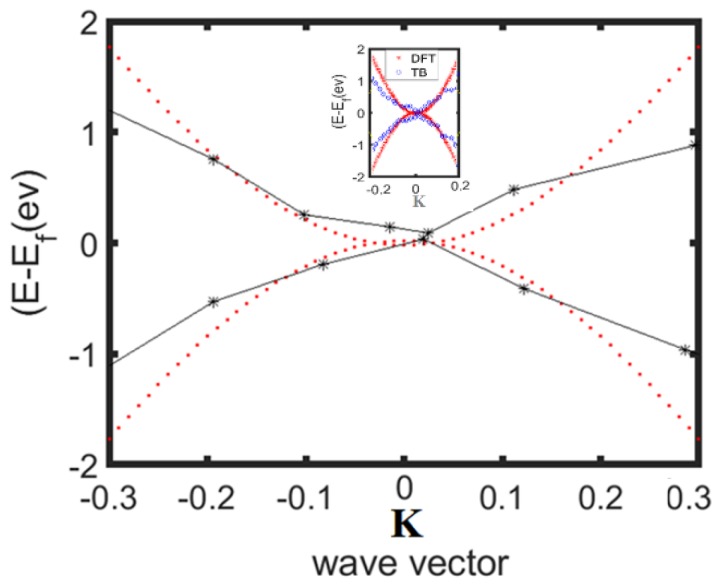
The comparison between the presented model (red dots) and density functional theory (DFT) simulation (black stars).

The result of the simulated structure is closer to the results of Figure 5 in response to the analytical model (comparison between Figure 5 and Figure 6), which indicates that the band structure is about 0.167 ev. The comparison study between the presented model and DFT simulation result is carried out as shown in Figure 7.

An acceptable agreement—especially on the k point—is detected, which confirms the accuracy of the proposed model. Therefore, the proposed analytical calculation could represent the prediction of electrical performance of BC_2_N, which also provides very fast modeling and simulation tools for band-gap investigation. On the other hand, the lattice parameter effect on the BC_2_N gap performance can be explored by the overlap energy gradient due to the stress and strain associated with the temperature or device fabrication limits. 

## 4. Conclusions

BC_2_N is one of the most stable structures of C_X_ (BN) y configuration. This structure is an in-between combination of hexagonal graphene and hexagonal boron nitride. The calculations showed that the band-gap energy of C_X_ (BN) y is between pristine graphene with a band gap of zero and boron nitride with a band-gap energy of 5.5 eV. The band gap firmly depends on either the formation energy or binding energy. Conceding and doping in the graphene sheet with boron and nitrogen atoms firmly depends on its location and amount, which affects its stability. In the present study, we focused on the amazing and notable structures of the BC_2_N family. In this structure, no bonding is found between C–C atoms, which caused the inspiration of unique electronic and optical properties as well as its application in designing electronic devices. This study focused on the dispersion relation investigation using an analytical calculation based on the nearest neighbor’s tight-binding method. This method supposes that the band gap of the main structure of BC_2_N alters from 2.41 in E_1_ to 1.12 in E_2_ and from 0.7182 eV to 1.3254 eV in E_4_. On the other hand, using figured alternating wave vector versus energy, it is found that the changes in overlap energy between boron–carbon and nitrogen–carbon can engender the band gap.
